# Methylmalonic Acidemia with Novel* MUT* Gene Mutations

**DOI:** 10.1155/2017/8984951

**Published:** 2017-10-12

**Authors:** Inusha Panigrahi, Savita Bhunwal, Harish Varma, Simranjeet Singh

**Affiliations:** Department of Pediatrics, Advanced Pediatric Centre, PGIMER, Chandigarh, India

## Abstract

A 5-year-old boy presented with recurrent episodes of fever, feeding problems, lethargy, from the age of 11 months, and poor weight gain. He was admitted and evaluated for metabolic causes and diagnosed as having methylmalonic acidemia (MMA). He was treated with vit B12 and carnitine supplements and has been on follow-up for the last 3 years. Mutation analysis by next generation sequencing (NGS), supplemented with Sanger sequencing, revealed two novel variants in the* MUT* gene responsible for MMA in exon 5 and exon 3, respectively. Recently he developed dystonic movements including orofacial dyskinesia. With advent of NGS, judicious use of NGS with Sanger sequencing can help identify causative possibly pathogenic mutations.

## 1. Case Presentation

**Figure 1:**
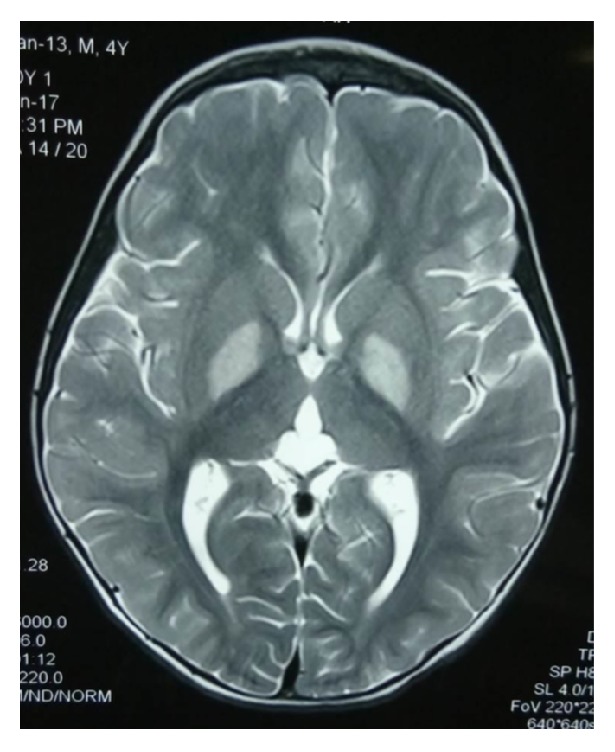
The MRI brain in the child with MUT-related MMA showing predominant frontoparietal abnormalities in form of encephalomalacia and gliosis.

The child presented for the first time at the age of 11 months, with complaints of fever, vomiting, poor feeding, and lethargy. On examination he had pallor and tachypnea and was drowsy. Further evaluation was suggestive of high anion-gap metabolic acidosis with ketonuria (urine ketones- 3+) with normal electrolytes, blood sugar (94 mg/dl), vitamin B12, and homocysteine. Plasma ammonia was 118 units, and plasma lactate was 2.9 units. TMS was normal but urine GC-MS revealed elevated 3-OH propionic acid [12.39 retention time (RT)] as well as elevated methyl malonic acid [16.92 RT, Suppl Figure 1, in Supplementary Material available online at https://doi.org/10.1155/2017/8984951]. Since then this child was on low protein diet, carnitine, biotin, thiamine, and vitamin B12 injections. Child was thereafter admitted on multiple occasions (7 times) with acute decompensation and managed as per protocol. Mutational analysis was sent for MMA which showed single heterozygous missense variant c.976 A>G (p.Arg326Gly) in exon 5 of* MUT* gene (genomic coordinates: chr 6: 49421405) as a variant of uncertain significance. Chromosomal microarray analysis done did not reveal any major deletion or duplication which could disrupt the gene. Since exon 3 and exon 6 were not adequately covered by NGS, further evaluation by Sanger sequencing for targeted exons was done and a 2nd mutation in exon 3 c.753 G>A (p.=) was identified. The variants were found to be damaging on SIFT database score (Suppl data). They were also predicted to be deleterious on Polyphen-2 and Mutation Taster and not found in the ExAC database. MRI brain done at the age of 4 years was showing multifocal cystic encephalomalacic changes with surrounding gliosis in deep white matter predominantly in frontoparietal regions (Figure 1).

In latest admission child was found to have fresh neurological findings in the form of perioral tremors, generalised hypertonia, and generalised dystonia with clonus with exaggerated deep tendon reflexes. He was treated with intravenous dextrose and sodium bicarbonate and was continued on carnitine and injection of vitamin B12. Plasma ammonia was 18 units and lactate level was 4.9 units. MRI brain was repeated and revealed bilateral basal ganglia hyperintensities suggestive of metabolic stroke. After the subsidence of acute crisis he was discharged on carnitine, injection of vitamin B12, and trihexyphenidyl. Parents were counseled regarding prognosis and for prenatal diagnosis next pregnancy.

## 2. Discussion

MMA presents with lethargy, acidosis, hypoglycemia/hyperglycemia, ketosis, and recurrent episodes. MMA due to* MUT* gene mutations usually led to severe phenotype, and around 35–40% of cases are due to new mutations [1, 2]. There can be missense or nonmutations, deletions, insertions, and so on leading to clinical phenotype. The advent of NGS technology has enabled better characterization of mutations in several populations. However, Sanger sequencing remains useful adjunct in molecular testing of these cases. Sometimes in NGS, due to incomplete coverage of the exons, Sanger sequencing is required to find mutations, if there is strong clinical suspicion. By careful use of both techniques, we could find the two variants responsible for the clinical condition. In a Saudi study on 60 patients of MMA, nonsense, missense, and frameshift mutations were detected across the* MUT* gene [3]. Another study in 43 Chinese patients identified 8 recurrent mutations and 10 novel mutations [4]. A previous Indian study in 15 patients of clinically diagnosed MMA identified one novel exon 12 mutation in* MUT* gene with predicted pathogenicity. Here, we identified two novel variants, one in exon 3 and another in exon 5 of the* MUT* gene. Both were labelled as variants of unknown significance (VUS). The exon 3 variant is a synonymous variant, and a different nucleotide change c.753 G>C (p.Lys251Asn) has been reported earlier in ClinVar. Some synonymous variants can also affect the splicing or protein function and lead to clinical phenotypes. The identified exon 5 variant is new, but another close variant c.977 G>A (p.Arg326Lys) has been reported in ClinVar. The variants were found to be deleterious on bioinformatic analysis and were not found in ExAC database. Both variants identified in present case possibly explain the phenotype of MMA in the child.

*MUT*-related MMA has poor prognosis in most cases. Specialised diet and supplements may not improve outcomes, even if diagnosed early. Early recognition and appropriate treatment of acute crises are necessary. Metabolic stroke can sometimes occur in the absence of acute metabolic decompensation, so meticulous neurological examination at each visit is useful. The options for therapy include early liver transplantation [5] and possibly gene therapy in the future. Genetic counseling and prenatal diagnosis help these families in making reproductive decisions.

## Supplementary Material

Suppl Fig 1: Urine GC-MS in the child showing elevated methylmalonic acid, lactate and few other metabolites.
