# Supplementary material for: Methylmalonic Acidemia with Novel MUT Gene Mutations
Source: Case Rep Genet. 2017 Oct 12;2017:8984951. doi: 10.1155/2017/8984951 (PMC5660767; doi:10.1155/2017/8984951)
Supplement: Supplementary file 1 — Suppl Fig 1: Urine GC-MS in the child showing elevated methylmalonic acid, lactate and few other metabolites. [file 8984951.f1.doc]

**Suppl Fig 1**

**And**

**SIFT score information**

Suppl Fig 1: Urine GC_MS in the child showed MMA as the dominant metabolite with significantly increased levels.


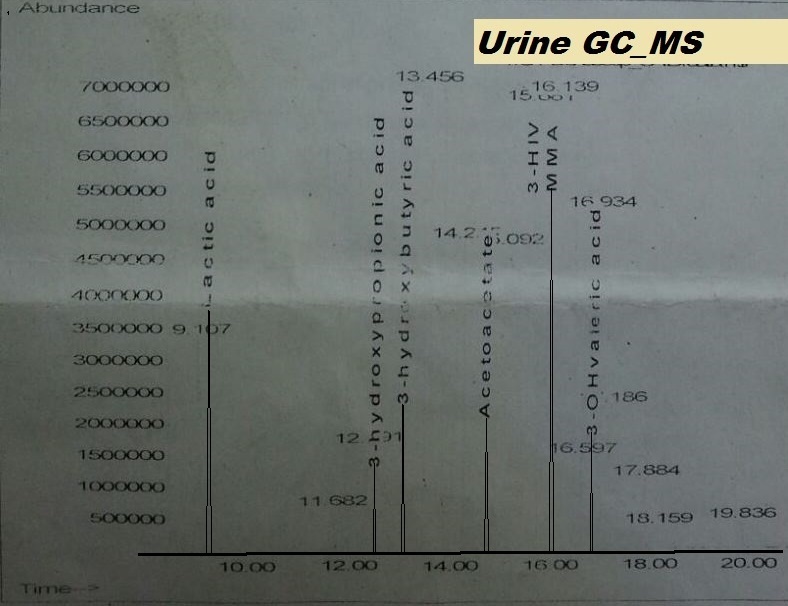


| **SIFT score_MMA variants_25/07/2017**  Job | Job size | Job ID | Job status | View results | Download results |
| --- | --- | --- | --- | --- | --- |
| Partitioned set 1 | Input rows 1 to 2 | 5e9ba4831e | Complete | [5e9ba4831e table](http://siftdna.org/sift-bin/catfile.csh?/mnt3/tmp//5e9ba4831e_nssnv_predictions.html) | [5e9ba4831e results](http://siftdna.org/www/sift/tmp/5e9ba4831e_nssnv_predictions.tsv) |
| Complete set | Input rows 1 to 2 | 736128bdc5 | Complete | [736128bdc5 table](http://siftdna.org/sift-bin/catfile.csh?/mnt3/tmp//736128bdc5_nssnv_predictions.html) | [736128bdc5 results](http://siftdna.org/www/sift/tmp/736128bdc5_nssnv_predictions.tsv) |

Batch Report

Number of input (non-intronic) variants: 2
Coding variants: 100% (2 out of 2)
Coding variants predicted: 100% (2 out of 2)
Tolerated: 0% ( out of 2)
Damaging: 100% (2 out of 2)

Novel: 100% (2 out of 2)
